# Supplementary material for: LhANS-rr1, LhDFR, and LhMYB114 Regulate Anthocyanin Biosynthesis in Flower Buds of Lilium ‘Siberia’
Source: Genes (Basel). 2023 Feb 23;14(3):559. doi: 10.3390/genes14030559 (PMC10048704; doi:10.3390/genes14030559)
Supplement: Supplementary file 1 [file genes-14-00559-s001.zip › Supplement Table S1.pdf]

Table 1. PCR primers used in the study.

Primer	Sequence（5'-3'）	Usage
*LD*F	ATGGAGAATGTGAAAGGCCCCGTG	Gene cloning for *LhDFR*
*LD*R	TTACTGAAGAGCAACGGAGACTTGT	
*LAc*F	ATGCCGACCGAGATCATGCCGTTGC	Gene cloning for *LhANS-rr1*
*LAc*R	TCACTTGAGAGAAGTGAAGTCCTCC	
*M14*F	ATGAGGAAGACGCCTCGCACAAAGAG	Gene cloning for *LhMYB114*
*M14*R	TTAATCTCCAGTTATTAAATCAGAAAG	
*CH1*F	AGTTCATCACCTTCACTGCCATCG	qRT-PCR for CHI
*CH1*R	CGGAGTATTGTTGCCCAGTCAGTT	
*F3*H	TTCTTGTGCCCAACGATGTCCCTG	qRT-PCR for F3H
*F3*R	GCCATGACATTCGTGCCTTCTCCT	
*CC1*F	ACGACAACACGCTCTGGAATGG	qRT-PCR for CCoAOMT-1
*CC1*R	GGCAGGCAGCAACATATCACTT	
*C3*F	ACTGCTGTCGTCAAAGAGTCCCT	qRT-PCR for C3H
*C3*R	CCAACGGGTTCTTCCACGCATT	
*CH*F	GCATCACCAAGAGCGAGCATCTC	qRT-PCR for CHS
*CH*R	TACCATATCCTGGCGAGCGTCAAG	
*AN*F	GGTGAACAAGGAGAGGGTCAGGAT	qRT-PCR for LhANS-rr1
*AN*R	ATGCTGGATGTGCTGCTTGAAAGT	
*CC2*F	CGATGCCGACAAGGACAACTACC	qRT-PCR for CCoAOMT-2
*CC2*R	TCAGCCGCCAATGCCTTATTCAG	
*CC3*F	CGATGCCGACAAGGACAACTACCT	qRT-PCR for CCoAOMT-3
*CC3*R	CCACCACAGAGCCATTCCAGAGTG	
*C41*F	TGCTGCCATTGAAACAACCCTCTG	qRT-PCR for C4H
*C41*R	TGTCGGGCTCAGTGATTTGGACTC	
*HC*F	CAATGAAGTACACCGCCGCTACC	qRT-PCR for HCT
*HC*R	GCTGCTGAACTTGCTGTCTGAGG	
*C4*F	CGATGCCGACAAGGACAACTACC	qRT-PCR for CCoAOMT-4
*C4*R	TCAGCCGCCAATGCCTTATTCAG	
*Ch2*F	ATGGATACACGGTCCGGGCTACTG	qRT-PCR for LhDFR
*Ch2*R	AACATGGTACACACCGGAGCAACC	
*C5*F	CGATGCCGACAAGGACAACTACC	qRT-PCR for CCoAOMT-5
*C5*R	TCAGCCGCCAATGCCTTATTCAG	
*C6*F	CGATGCCGACAAGGACAACTACC	qRT-PCR for CCoAOMT-6
*C6*R	TCAGCCGCCAATGCCTTATTCAG	
*4C*F	GATTGTCGTGGAGCTGGCGAAGAG	qRT-PCR for 4CL
*4C*R	GTCATCCCGTAACCCTGCCCAAGT	

*UD*F	CTCACGGCGTCAAGACCACCATT	qRT-PCR for UDPG
*UD*R	ACGTTCTCGCAGCCTTCAGGAAG	
*LhActin*F	GCATCACACCTTCTACAACG	Reference gene
*LhActin*R	GAAGAGCATAACCCTCATAGA	
*MTR*F	TGAGTAAGGTTACCGGAATTCTGGCAATACTTCTGATTCGTCCTC	Fragment amplification for TRV of *LhMYB114*
*MTR*R	GGGACATGCCCGGGCCTCGAGCATCTCAATGTACCGTGCTTCTCT	
*Lav*F	TGAGTAAGGTTACCGGAATTCCGACGAAGCCTTGAAGTTGAAC	Fragment amplification for TRV of *LhANS-rr1*
*Lav*R	GGGACATGCCCGGGCCTCGAGCCAATCCTCTGCTGCCTTCTT	
*LDv*F	TGAGTAAGGTTACCGGAATTCGTGCTAGTGGCTATGTTGGTT	Fragment amplification for TRV of *LhANS-rr1*
*LDv*R	GGGACATGCCCGGGCCTCGAGGCTCTCATCCTCGCTAAGATC	
