# Supplementary material for: LhANS-rr1, LhDFR, and LhMYB114 Regulate Anthocyanin Biosynthesis in Flower Buds of Lilium ‘Siberia’
Source: Genes (Basel). 2023 Feb 23;14(3):559. doi: 10.3390/genes14030559 (PMC10048704; doi:10.3390/genes14030559)
Supplement: Supplementary file 1 [file genes-14-00559-s001.zip › Supplementary technical terms.pdf]

Technical Terms:
6 CCoAOMT (caffeoyl-CoA3-methyl-transferase),
UDPG (pyrophosphorylase),
HCT（Hydroxycinnamoyltransferase）,
C4H (cinnamate 4-hydroxylase),
4CL (4-coumaroyl-coenzyme A ligase),
CHS (chalcone synthase),
CHI (chalcone ismoerase),
F3H (flavonoid 3-hydroxylase),
LhDFR (Lh-dihydroflavonol 4-reductase),
LhANS-rr1 (Lh-anthocyanidin synthase),
and UFGT (UDPG-flavonoid-3-O-glycosyltranferase).
