# Supplementary figures and images for: LhANS-rr1, LhDFR, and LhMYB114 Regulate Anthocyanin Biosynthesis in Flower Buds of Lilium ‘Siberia’
Source: Genes (Basel). 2023 Feb 23;14(3):559. doi: 10.3390/genes14030559 (PMC10048704; doi:10.3390/genes14030559)

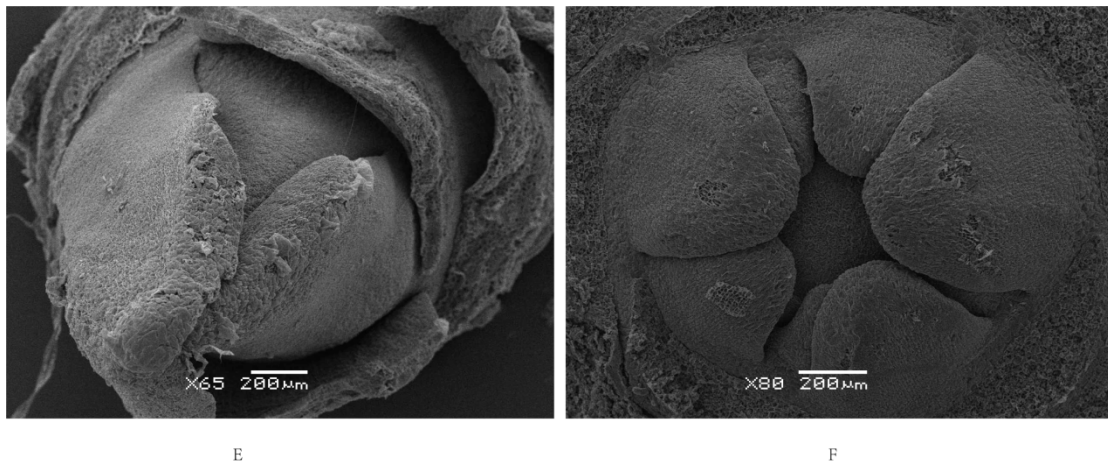

Figure S1. The microscopic view of epical meristem of Lilium hybrid 'Siberia' at E and F stage.

Supplement: Supplementary file 1 [file genes-14-00559-s001.zip › Supplementary picture1.pdf]
